# ﻿Two new saprobic species of *Helicosporium* and *Dictyosporium* and a new host record of *Neohelicomyces guizhouensis* isolated from *Phellodendron amurense* (Rutaceae, Tracheophyta) in China

**DOI:** 10.3897/mycokeys.127.175758

**Published:** 2026-01-07

**Authors:** Shi-Ping Zou, Yuan-Pin Xiao, Qiu-Yuan Tang, Yu-Bi Chen, Yong-Zhong Lu, Ning-Guo Liu, Dan-Feng Bao, Ratchadawan Cheewangkoon

**Affiliations:** 1 Department of Entomology and Plant Pathology, Faculty of Agriculture, Chiang Mai University, Chiang Mai 50200, Thailand Chiang Mai University Chiang Mai Thailand; 2 School of Food and Pharmaceutical Engineering, Guizhou Institute of Technology, Guiyang 550003, China Guizhou Institute of Technology Guiyang China; 3 Guizhou Industry Polytechnic College, Guiyang 551400, China Guizhou Industry Polytechnic College Guiyang China; 4 Guizhou Key Laboratory of Agricultural Microbiology, Guizhou Academy of Agricultural-Sciences, Guiyang 550009, China Guizhou Academy of Agricultural-Sciences Guiyang China; 5 Engineering Research Center of the Utilization for Characteristic Bio-Pharmaceutical Resources in Southwest, Ministry of Education, Guizhou University, Guiyang 550025, China Guizhou University Guiyang China

**Keywords:** Cheirosporous, helicosporous, phylogeny, taxonomy, two new species

## Abstract

During a survey of saprobic fungi associated with the medicinal plant *Phellodendron amurense* in Guizhou Province, China, three taxa were isolated and examined. Based on morphological characteristics and multigene phylogenetic analyses, two novel species, *Dictyosporium phellodendri* (Dictyosporiaceae, Pleosporales) and *Helicosporium phellodendri* (Tubeufiaceae, Tubeufiales), are proposed. In addition, *Neohelicomyces guizhouensis* (Tubeufiaceae, Tubeufiales) is reported as a new host record. Detailed morphological descriptions, illustrations and molecular evidence are provided to support the establishment of these taxa.

## ﻿Introduction

*Phellodendron amurense* (Rutaceae, Tracheophyta), a deciduous plant, is mainly distributed in East Asia. It is rich in alkaloids, flavonoids, triterpenoids, coumarins and other bioactive compounds (Chen et al. 2024). In China, it is renowned as one of the most important traditional Chinese medicine plants, listed in the Chinese Pharmacopoeia (2020 edition) and has been widely used for thousands of years (Xian et al. 2014; Wang et al. 2015; Dulan et al. 2022; Zhang et al. 2024). Medicinal plants are recognised as potential reservoirs for uncovering fungal diversity (Du et al. 2024). Endophytic and pathogenic fungi associated with *P. amurense* have been documented (Cao and Qi 1990; Li 2006; Li et al. 2009, 2018; Wen et al. 2022). However, saprobic fungi occurring on this medicinal host remain poorly explored.

Tubeufiaceae was established by Barr (1979) to accommodate the type genus *Tubeufia* and comprises mainly saprobic taxa inhabiting decaying wood. Members of the family are widely distributed in tropical and subtropical regions (Rossman 1987; Kirk et al. 2001; Lumbsch et al. 2010; Boonmee et al. 2014; Luo et al. 2017; Lu et al. 2018a, b), occurring in both terrestrial wood and aquatic environments (Hyde et al. 2016, 2017; Brahamanage et al. 2017; Chaiwan et al. 2017; Lu et al. 2017a, b, c, 2018a, b; Liu et al. 2018; Hongsanan et al. 2020). Morphologically, members of the family are characterised by helicosporous conidia in the asexual morph (Linder 1929; Samuels and Muller 1979; Spatafora et al. 2006;Tsui et al. 2006; Tsui Barr Lu et al. 2018b; Dong et al. 2020;Zhang et al.2020) and by superficial ascomata, bitunicate asci and hyaline to pale brown, elongate to obovoid, septate ascospores in the sexual morph (Barr 1980; Kodsueb et al. 2006; Boonmee et al. 2011, 2014; Brahamanage et al. 2017; Lu et al. 2018b; Karimi et al. 2025; Sun et al. 2025). Currently, Tubeufiaceae comprises 54 genera (Lu et al. 2018b; Liu et al. 2019a, b, c, d; Wijayawardene et al. 2022; Karimi et al. 2025), amongst which *Helicosporium* and *Neohelicomyces* represent common helicosporous hyphomycetes within the order Tubeufiales (Lu et al. 2018b).

Dictyosporiaceae was introduced by Boonmee et al (2016) to accommodate most cheirosporous hyphomycetous genera, with *Dictyosporium* designated as the type genus. According to Wang et al (2025), the family includes 23 genera and 178 species, excluding one novel species proposed in this study. Members of Dictyosporiaceae are saprobes on plant litter and decaying wood in both terrestrial and aquatic habitats worldwide (Boonmee et al. 2016; Fu et al. 2021; Shen et al. 2022). The type genus *Dictyosporium* is characterised by its cheiroid conidia (Corda 1836).

During a taxonomic survey of saprophytic fungi, associated with *Phellodendron amurense* in Guizhou Province, China, six fresh collections were obtained from the decaying branches in two forests sites. Morphological observations combined with multigene phylogenetic analyses revealed two novel species, *Helicosporium phellodendri* and *Dictyosporium phellodendri* and a new host record, *Neohelicomyces guizhouensis*. Detailed morphological descriptions, illustrations and molecular evidence are provided herein.

## ﻿Materials and methods

### ﻿Sample collection, specimen examination and isolation

Fresh specimens were collected from decaying wood of *Phellodendron amurense* in Anshun City and Guiyang City, Guizhou, China, in October 2024. Relevant collection data were recorded in the field. Specimens were examined and observed using a stereomicroscope (SMZ-168, Nikon, Japan). Micromorphological features were observed and photographed with an ECLIPSE Ni compound microscope (Nikon, Tokyo, Japan), equipped with a Canon 90D digital camera. Photoplates were prepared using Adobe Photoshop CC 2019 (Adobe Systems, USA) and Tarosoft® Image Frame Work.

Single spore isolations were performed following the method described by Senanayake et al. (2020). Germinated conidia were transferred on to fresh potato dextrose agar (PDA) plates and incubated in a 25 °C for colony development.

### ﻿Material deposition

Dried specimens were deposited in the Herbarium of Cryptogams Kunming Institute of Botany, Academia Sinica (HKAS), Kunming, China. Living cultures were deposited in the Guizhou Culture Collection (GZCC), Guizhou, China. New taxa were registered on the Index Fungorum database (Index Fungorum 2025).

### ﻿DNA extraction, PCR amplification and sequencing

Fresh mycelia were scraped using a sterile toothpick and transferred to 1.5 ml microcentrifuge tube. Genomic DNA was extracted using the Trelief Hi-Pure Fungus Genomic DNA Extraction Kit (Tsingke, China) according to the manufacturer’s instructions. Four genetic loci (LSU, ITS, *tef*1-α and *rpb*2) were amplified using the primers LR0R/ LR5 (Vilgalys and Hester 1990), ITS5/ ITS4 (White et al. 1990), EF1-983F/EF1-2218R (Rehner and Buckley 2005) and fRPB2-5F/fRPB2-7cR (Liu et al. 1999), respectively. PCR amplification was conducted in a 25 μl reaction mixture containing 21 μl of 1.1 × T3 Super PCR Mix (Tsingke Biotech, Chongqing, China), 2 μl of DNA template and 1 μl of each primer. The cycling parameters for LSU, ITS, *tef*1-a and *rpb*2 followed the protocols of Ma et al. (2024b). Purified PCR products were sequenced by Tsingke Biological Engineering Technology and Services Co., China.

### ﻿Phylogenetic analyses

Sequence quality was verified using BioEdit v. 7.0.5.3 (Hall 1999). Forward and reverse reads were assembled with SeqMan v. 7.0.0 (DNASTAR, Madison, WI, USA; Swindell and Plasterer 1997) and deposited in GenBank. Similar sequences were retrieved using the BLASTn tool in NCBI (https://www.ncbi.nlm.nih.gov/). Reference sequences used for phylogenetic analyses are listed in Tables 1, 2. Each locus was aligned using MAFFT v.7.473 (Katoh et al. 2019) on the online server (https://mafft.cbrc.jp/alignment/server/) and ambiguous regions were trimmed using trimAl v.1.2rev59 (Capella-Gutiérrez et al. 2009). The alignments were concatenated using SequenceMatrix-Windows-1.7.8 (Vaidya et al. 2011).

**Table 1.** Taxa used for Tubeufiaceae species in this study and their GenBank accession numbers.

Taxon	Strain	GenBank Accession numbers			
		LSU	ITS	*tef*1–α	*rpb*2
*Acanthostigma chiangmaiensis*	MFLUCC 10-0125^T^	JN865197	JN865209	KF301560	–
*Acanthostigma perpusillum*	UAMH 7237	AY856892	AY916492	–	–
*Botryosphaeria agaves*	MFLUCC 10-0051	JX646807	JX646790	–	–
*Botryosphaeria dothidea*	CBS 115476	DQ678051	KF766151	DQ767637	DQ677944
*Helicosporium acropleurogenum*	CGMCC 3.25563^T^	PP639430	PP626574	PP596333	PP596460
*Helicosporium aquaticum*	MFLUCC 17-2008^T^	MH558859	MH558733	MH550924	MH551049
*Helicosporium brunneisporum*	CGMCC 3.25542^T^	PP639433	PP626577	PP596336	PP596463
*Helicosporium changjiangense*	GZCC 22-2113^T^	PP639434	PP626578	PP596337	PP596464
*Helicosporium flavisporum*	MFLUCC 17-2020^T^	MH558860	MH558734	MH550925	MH551050
*Helicosporium flavum*	MFLUCC 16-1230^T^	KY873621	KY873626	KY873285	–
*Helicosporium hainanense*	GZAAS 22-2006^T^	OP508770	OP508730	OP698081	OP698070
*Helicosporium jiangkouense*	HKAS 128933^T^	PP639436	PP626580	PP596339	PP596466
*Helicosporium latisporum*	HKAS 128960^T^	PP639437	PP626582	PP596340	PP596467
*Helicosporium liuzhouense*	GZCC 22-2014^T^	OQ981402	OQ981394	OQ980476	OQ980474
*Helicosporium luteosporum*	MFLUCC 16-0226^T^	KY321327	KY321324	KY792601	MH551056
*Helicosporium multidentatum*	GZCC 22-2013^T^	OQ981403	OQ981395	OQ980477	OQ980475
*Helicosporium multiseptatum*	HKAS_136882 ^T^	PQ570860	PQ570843	PQ761135	–
*Helicosporium nanningense*	GZCC 22-2175^T^	OR066425	OR066418	OR058864	OR058857
***Helicosporium phellodendri***	**GZCC 25-0005^T^**	**PX220124**	**PX220122**	**PX667152**	**PX667156**
***Helicosporium phellodendri***	**GZCC 25-0006**	**PX220125**	**PX220123**	**PX667153**	**PX667157**
*Helicosporium ramosiphorum*	CGMCC 3.25541^T^	PP639432	PP626576	PP596335	PP596462
*Helicosporium rubrum*	MFLUCC 24-0090^T^	PQ098514	PQ098477	PQ490681	PQ490675
*Helicosporium setiferum*	MFLUCC 17-1994^T^	MH558861	MH558735	MH550926	MH551051
*Helicosporium sexuale*	MFLUCC 16-1244^T^	MZ538537	MZ538503	MZ567082	MZ567111
*Helicosporium* sp.	NBRC 9014	AY856903	AY916489	–	–
*Helicosporium thailandense*	MFLUCC 18-1407^T^	MN913718	MT627698	MT954371	–
*Helicosporium vegetum*	GZCC 23-0060	PP639439	PP626584	PP596342	PP596469
*Helicosporium vesicarium*	MFLUCC 17-1795^T^	MH558864	MH558739	MH550930	MH551055
*Helicosporium viridiflavum*	MFLUCC 17-2336^T^	–	MH558738	MH550929	MH551054
*Helicosporium viridisporum*	GZCC 22-2008^T^	OP508776	OP508736	OP698087	OP698076
*Helicotubeufia hydei*	MFLUCC 17-1980^T^	MH290026	MH290021	MH290031	MH290036
*Helicotubeufia jonesii*	MFLUCC 17-0043^T^	MH290025	MH290020	MH290030	MH290035
*Muripulchra aquatica*	MFLUCC 15-0249^T^	KY320549	KY320532	–	–
*Neohelicomyces acropleurogenus*	CGMCC 3.25549^T^	PP639450	PP626594	PP596351	PP596478
*Neohelicomyces aquaticus*	MFLUCC 16-0993^T^	KY320545	KY320528	KY320561	MH551066
*Neohelicomyces aseptatus*	CGMCC 3.25564^T^	PP639451	PP626595	PP596352	PP596479
*Neohelicomyces dehongensis*	MFLUCC 18-1029^T^	MN913709	NR_171880	MT954393	–
*Neohelicomyces denticulatus*	GZCC 19-0444^T^	MW133855	OP377832	–	–
*Neohelicomyces deschampsiae*	CPC 33686^T^	MK442538	MK442602	–	–
*Neohelicomyces edgeworthiae*	CGMCC 3.25565^T^	PP639453	PP626597	PP596354	PP596481
*Neohelicomyces grandisporus*	KUMCC 15-0470^T^	KX454174	KX454173	–	MH551067
*Neohelicomyces guizhouensis*	GZCC 23-0725^T^	PP512973	PP512969	PP526727	PP526733
***Neohelicomyces guizhouensis***	**GZCC 25-0007**	**PX645246**	**PX649070**	**PX667150**	**PX667154**
***Neohelicomyces guizhouensis***	**GZCC 25-0008**	**PX645247**	**PX649071**	**PX667151**	**PX667155**
*Neohelicomyces guttulatus*	CGMCC 3.25550^T^	PP639454	PP626598	PP596355	–
*Neohelicomyces hainanensis*	GZCC 22-2009^T^	OP508774	OP508734	OP698085	OP698074
*Neohelicomyces helicosporus*	GZCC 23-0633^T^	PP512975	PP512971	PP526729	PP526735
*Neohelicomyces hyalosporus*	GZCC 16-0086^T^	MH558870	MH558745	MH550936	MH551064
*Neohelicomyces hydei*	GZCC 23-0727^T^	PP512977	–	PP526731	PP526737
*Neohelicomyces lignicola*	CGMCC 3.25551^T^	PP639456	PP626600	PP596357	PP596483
*Neohelicomyces longisetosus*	NCYU-106H1-1-1^T^	–	MT939303	–	–
*Neohelicomyces macrosporus*	CGMCC 3.25552^T^	PP639457	PP626601	PP596358	PP596484
*Neohelicomyces maolanensis*	GZCC 23-0079^T^	PQ098529	–	PQ490683	PQ490677
*Neohelicomyces melaleucae*	CPC 38042^T^	MN567661	MN562154	MN556835	–
*Neohelicomyces pallidus*	CBS 271.52	AY856887	AY916461	–	–
*Neohelicomyces pallidus*	CBS 962.69	AY856886	AY916460	–	–
*Neohelicomyces pandanicola*	KUMCC 16-0143^T^	MH260307	MH275073	MH412779	–
*Neohelicomyces qixingyaensis*	CGMCC 3.25569^T^	PP639458	PP626602	PP596359	PP596485
*Neohelicomyces submersus*	MFLUCC 16-1106^T^	KY320547	KY320530	–	MH551068
*Neohelicomyces subtropicus*	GZCC 23-0076^T^	PQ098530	PQ098492	PQ490685	PQ490679
*Neohelicomyces thailandicus*	MFLUCC 11-0005^T^	MN913696	NR_171882		–
*Neohelicomyces xiayadongensis*	CGMCC 3.25568^T^	PP639460	PP626604	PP596361	PP596487
*Neohelicomyces yunnanensis*	GZCC 23-0735^T^	PP664113	PP664109	–	–
*Tubeufia guttulata*	GZCC 23-0404^T^	OR030834	OR030841	OR046678	OR046684
*Tubeufia hainanensis*	GZCC 22-2015^T^	OR030835	OR030842	OR046679	OR046685
*Tubeufia javanica*	MFLUCC 12-0545^T^	KJ880036	KJ880034	KJ880037	–
*Tubeufia krabiensis*	MFLUCC 16-0228^T^	MH558917	MH558792	MH550985	MH551118
*Tubeufia latispora*	MFLUCC 16-0027^T^	KY092412	KY092417	KY117033	MH551119
*Tubeufia laxispora*	MFLUCC 16-0232^T^	KY092408	KY092413	KY117029	MF535287
*Tubeufia mackenziei*	MFLUCC 16-0222^T^	KY092410	KY092415	KY117031	MF535288
*Tubeufia muriformis*	GZCC 22-2039^T^	OR030836	OR030843	OR046680	OR046686
*Tubeufia nigroseptum*	CGMCC 3.20430^T^	MZ853187	MZ092716	OM022002	OM022001
*Tubeufia pandanicola*	MFLUCC 16-0321^T^	MH260325	MH275091	OP698085	OP698074

Note: Newly-generated sequences are shown in bold. A dash (“–”) indicates data unavailable in GenBank and “^T^” denotes an ex-type strain.

**Table 2.** Taxa of Dictyosporiaceae used in this study and their GenBank accession numbers.

Taxon	Strain	GenBank Accession numbers		
		LSU	ITS	*tef*1–α
*Aquadictyospora clematidis*	MFLUCC 17-2080	MT214545	MT310592	MT394727
*Aquadictyospora lignicola*	MFLUCC 17-1318	MF948629	MF948621	MF953164
*Dictyosporium alatum*	ATCC 34953 ^T^	DQ018101	NR_077171	–
*Dictyosporium appendiculatum*	MFLUCC 17-2259 ^T^	MH376715	MH388343	–
*Dictyosporium aquaticum*	MF1318 ^T^	–	KM610236	–
*Dictyosporium australiense*	HKUCC 8797	–	DQ018092	–
*Dictyosporium bulbosum*	yone221	AB807511	LC014544	AB808487
*Dictyosporium cycadicola*	HUEST 24.0140	PP740388	PP740382	PP776570
*Dictyosporium digitatum*	KH 401	AB807515	LC014545	AB808491
*Dictyosporium digitatum*	yone 280	AB807512	LC014547	AB808488
*Dictyosporium duliujiangense*	KUNCC 23-15949	PQ309046	PQ309055	PQ346450
*Dictyosporium duliujiangense*	GZCC 19-0426 ^T^	MW133815	OQ842725	OQ850746
*Dictyosporium elegans*	FMR 13125	KY853501	KY853441	–
*Dictyosporium elegans*	NBRC 32502 ^T^	DQ018100	DQ018087	–
*Dictyosporium giganticum*	BCC 11346	–	DQ018095	–
*Dictyosporium guangdongense*	ZHKUCC 24-0002 ^T^	PP326213	PP326190	–
*Dictyosporium guttulatum*	MFLUCC 1- 0258 ^T^	MH376717	MH388345	MH388379
*Dictyosporium heptasporum*	CBS 396.59	–	DQ018090	–
*Dictyosporium hongkongensis*	KUMCC 17-0268	MH376718	MH388346	MH388380
*Dictyosporium hughesii*	K 1847	AB807517	LC014548	AB808493
*Dictyosporium hughesii*	KUNCC 23-15923	PQ309047	PQ309056	–
*Dictyosporium krabiense*	MFLU 16-1890 ^T^	MH376719	–	MH388381
*Dictyosporium licualae*	SNC182	–	PP594915	PP740420
*Dictyosporium meiosporum*	MFLUCC 10-0131 ^T^	KP710945	KP710944	–
*Dictyosporium muriformis*	GZCC 20-0006 ^T^	MN897834	MT002304	MT023011
*Dictyosporium nigroapice*	MFLUCC 17-2053 ^T^	MH381777	MH381768	MH388821
*Dictyosporium nigroapice*	BCC 3555	–	DQ018085	–
*Dictyosporium olivaceosporum*	KH 375 ^T^	AB807514	LC014542	AB808490
*Dictyosporium palmae*	CBS-H 22129	KX555648	–	–
*Dictyosporium pandanicola*	MFLU 16-1886 ^T^	MH376720	MH388347	MH388382
***Dictyosporium phellodendri***	**GZCC 25-0522 ^T^**	**PV910482**	**PV916337**	**PX667148**
***Dictyosporium phellodendri***	**GZCC 25-0523**	**PV910483**	**PV916338**	**PX667149**
*Dictyosporium sexualis*	MFLUCC 10-0127 ^T^	KU179106	KU179105	–
*Dictyosporium* sp.	J15	–	PP033644	–
*Dictyosporium* sp.	J16	–	PP033645	–
*Dictyosporium* sp.	KT 2025	–	MZ359876	–
*Dictyosporium* sp.	MFLUCC 15-0629	MH381775	MH381766	MH388819
*Dictyosporium* sp.	19VA07	–	JX270548	–
*Dictyosporium fluminicola*	KUNCC 23-17212	PQ226144	PQ038338	PQ227066
*Dictyosporium stellatum*	CCFC 241241 ^T^	JF951177	–	–
*Dictyosporium strelitziae*	CBS 123359 ^T^	FJ839653	–	–
*Dictyosporium tetrasporum*	K 2865	AB807519	LC014551	AB808495
*Dictyosporium thailandicum*	MFLUCC 13-0773 ^T^	KP716707	KP716706	–
*Dictyosporium toruloides*	CBS 209.65	DQ018104	DQ018093	–
*Dictyosporium tratense*	MFLUCC 17-2052 ^T^	MH381776	MH381767	MH388820
*Dictyosporium tubulatum*	MFLUCC 17-2056	MH381779	MH381770	–
*Dictyosporium tubulatum*	MFLUCC 15-0631 ^T^	MH381778	MH381769	MH388822
*Dictyosporium variabilisporum*	ZHKUCC 24-0003 ^T^	PP326215	PP326192	PP333112
*Dictyosporium wuyiense*	CGMCC 3.18703 ^T^	–	KY072977	–
*Dictyosporium zhejiangense*	HKAS 136886	PQ569961	PQ324184	–
*Dictyosporium zhejiangense*	MW–2009a ^T^	–	FJ456893	–

Note: Newly-generated sequences are shown in bold. A dash (“–”) indicates data unavailable in GenBank and “^T^” denotes an ex-type strain.

Maximum Likelihood (ML) analyses were performed with the IQ-TREE web server (http://iqtree.cibiv.univie.ac.at/, Nguyen et al. 2015; Zeng et al. 2023) using default parameters. Bayesian Inference (BI) analyses followed the procedures described by Lu et al (2022). The best-fit substitution model for each gene partition was determined using MrModelTest 2.3 under the Akaike Information Criterion (AIC) (Nylander et al. 2008).

### ﻿Phylogenetic results

**Figure 1.**
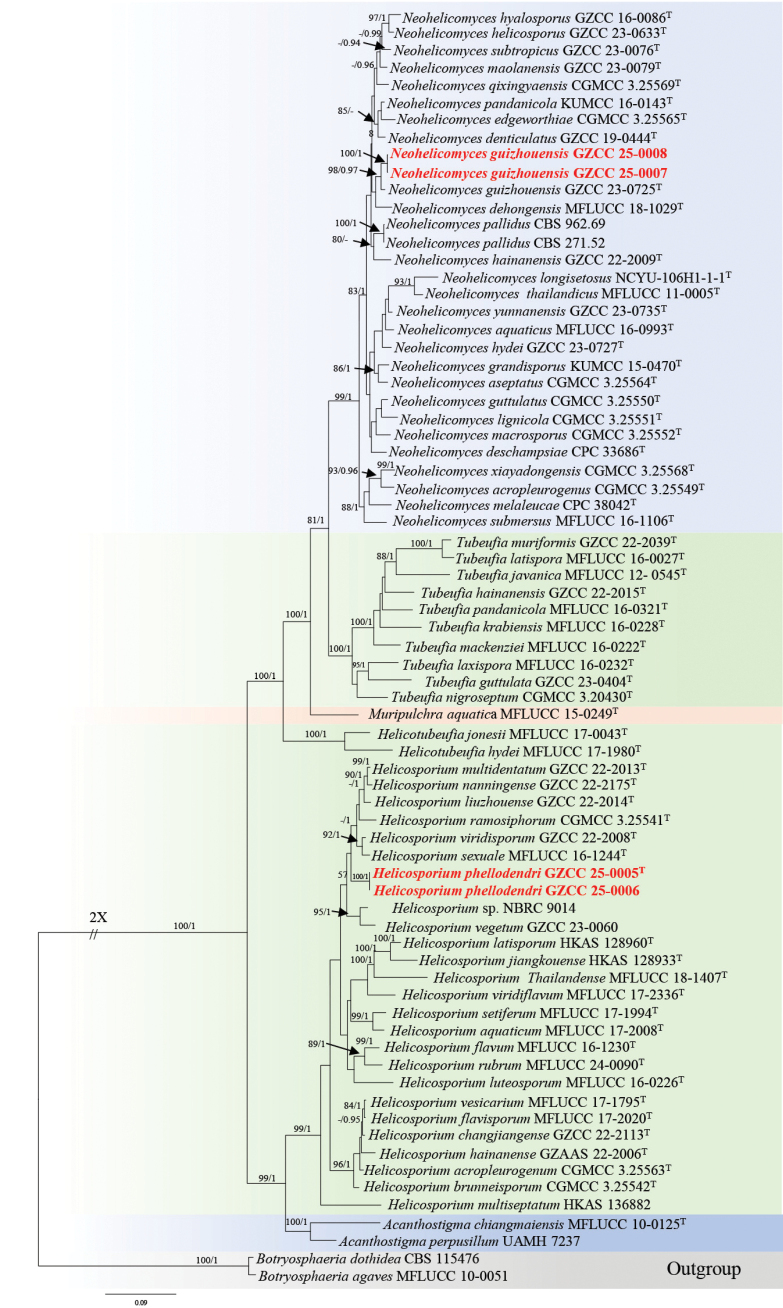
Maximum Likelihood (ML) phylogenetic tree, based on the combined dataset of LSU, ITS, *tef*1-α and *rpb*2 sequences. Bootstrap support values for ML analysis (≥ 75%) and posterior probability (PP) values from Bayesian inference (≥ 0.95) are shown at the corresponding nodes. Ex-type strains are indicated by “^T^” and newly-generated isolates are highlighted in bold red.

The first phylogenetic tree represents the phylogenetic positions of the new collections within Tubeufiaceae, based on four loci: LSU, ITS, *tef*1-α and *rpb*2.The concatenated dataset comprised 3,411 characters (LSU: 1–851, ITS: 852–1,441, *tef*1-α: 1,442–2,353, *rpb*2: 2,354–3,411), including gaps. A total of 73 strains, including isolates obtained in this study and two outgroups, *Botryosphaeria agaves* (MFLUCC 10-0051) and *B. dothidea* (CBS 115476), were analysed. Both ML and BI analyses yielded similar tree topologies. Fig. 1 illustrates the best- scoring ML tree, with a final likelihood value of -29709.023. In our phylogenetic tree (Fig. 1), our strains (GZCC 25-0007 and GZCC 25-0008) clustered within *Neohelicomyces*, while GZCC 25-0005 and GZCC 25-0006 clustered within *Helicosporium* in the family Tubeufiaceae.

**Figure 2.**
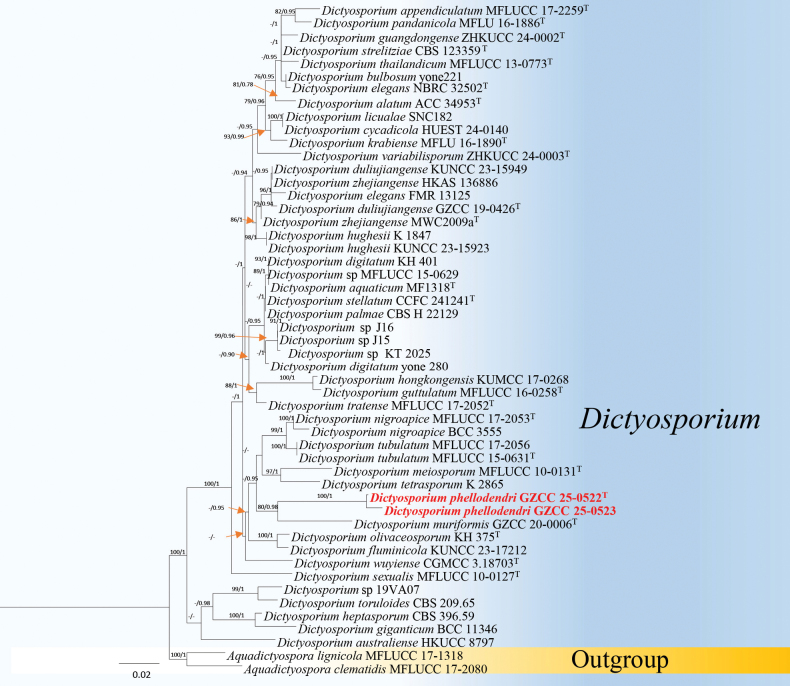
Maximum Likelihood (ML) phylogenetic tree, based on the combined dataset of LSU, ITS and *tef*1-α sequences. Bootstrap support values for ML analysis (≥ 75%) and posterior probability (PP) values from Bayesian inference (≥ 0.95) are shown at the corresponding nodes. Ex-type strains are indicated by “^T^” and newly-generated isolates are highlighted in bold red.

Phylogenetic analysis of *Dictyosporium* was based on three loci: LSU, ITS and *tef*1-α. The dataset comprised a total of 51 strains, which included isolates obtained in this study as well as the two outgroup taxa, *Aquadictyospora clematidis* (MFLUCC 17-2080) and *A. lignicola* (MFLUCC 17-1318). The concatenated sequence matrix contains 2,352 characters (LSU: 1–851, ITS: 852–1,442, *tef*1-α: 1,443–2,352) including gaps. The phylogenetic tree (Fig. 2) reveals that GZCC 25-0522 and GZCC 25-0523 cluster together with maximal support values (ML/PP = 100/1), forming a distinct and well-supported monophyletic lineage within *Dictyosporium*.

## ﻿Taxonomy

### ﻿ Helicosporium

Taxon classificationFungiTubeufialesTubeufiaceae

Nees, Syst. Pilze (Würzburg): 68 (1816) [1816–17]

62429C69-8BC8-5A47-9600-40500E1D39D3

#### Notes.

*Helicosporium*, one of the three earliest described helicosporous genera (*Helicoma*, *Helicomyces* and *Helicosporium*) (Link 1809; Nees 1817; Corda 1837), was established by Nees (1817) with *H. vegetum* as the type species. To date, 118 species are recorded in the Index Fungorum (2025). However, based on combined morphological and molecular evidence, only about 32 species are currently accepted in this genus (Lu et al. 2018b; Xiao et al. 2023; Ma et al. 2024b; Bai et al. 2025; Peng et al. 2025; Sun et al. 2025). Amongst these, sexual morphs have been confirmed for only five species: *H. flavum*, *H. sexuale*, *H. vegetum*, *H. rubrum* and *H. multiseptatum* (Boonmee et al. 2014, 2021; Brahmanage et al. 2017; Peng et al. 2025; Sun et al. 2025). Most species of *Helicosporium* are saprobes inhabiting freshwater and terrestrial environments, with a broad geographical distribution (Morgan-Jones and Goos 1992; Zhao et al. 2007; Lu et al. 2017a, 2018b, 2022; Dong et al. 2020; Boonmee et al. 2021; Hsieh et al. 2021; Xiao et al. 2023; Ma et al. 2024b). The majority of *Helicosporium* species have been isolated from decaying wood of unidentified plant hosts, while *H. multiseptatum* as the saprobe and *H. thailandicum* as the pathogen obtained from Poaceae sp. and *Elaeis* sp. (Arecaceae), respectively (Sun et al. 2025). In this study, we introduce a new *Helicosporium* species which was isolated from medicinal plants.

### ﻿ Helicosporium phellodendri

Taxon classificationFungiTubeufialesTubeufiaceae

S.P. Zou, Y. P. Xiao & Y.Z. Lu sp. nov.

3979AD23-9DE3-552F-8105-B96E019FAFEC

Index Fungorum: IF904507

Fig. 3

#### Etymology.

the specific epithet “*phellodendri*” refers to the host genus name “*Phellodendron*” from which the fungus was isolated.

#### Holotype.

HKAS 145879.

#### Description.

*Saprobic* on dead wood of *Phellodendron amurense*. ***Sexual morph*** Undetermined. ***Asexual morph*** Hyphomycetous, helicosporous. ***Colonies*** on natural substrate superficial, effuse, gregarious, with masses of crowded, glistening conidia, yellowish-green. ***Mycelium*** partly immersed, partly superficial, composed of pale brown to brown, branched, septate, guttulate, smooth hyphae. ***Conidiophores*** 89–97 × 4–5 μm (x̄ = 94 × 4.5 μm, n = 20), macronematous, mononematous, erect, cylindrical, straight or broadly curved, simple or occasionally branched, septate, brown at base, paler towards the apex, thick-walled. ***Conidiogenous cells*** 9.5–10.5 × 3.5–4.8 μm (x̄ = 10 × 4.3 μm, n = 15), holoblastic, monoblastic, integrated, intercalary, cylindrical, pale brown to brown, smooth-walled. ***Conidia*** solitary, pleurogenous, helicoid, tapering towards the ends, 10.5–12.5 μm diam. and conidial filament 1.5–2 μm wide (x̄ = 11.5 × 1.8 μm, n = 25), 60–65 μm long (x̄ = 63 μm, n = 25), tightly coiled 2½–3 times, not becoming loose in water, multi-septate, hyaline, smooth-walled.

**Figure 3.**
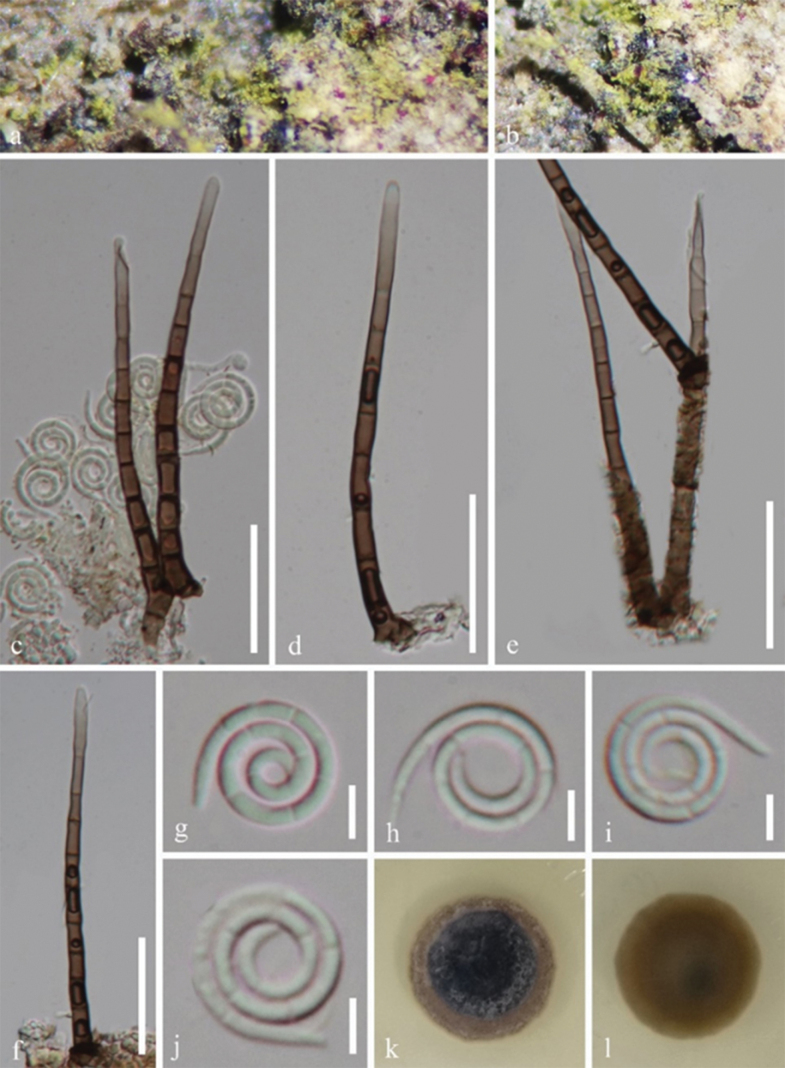
*Helicosporium phellodendri* (HKAS 145879, holotype). **a, b.** Colonies on the natural substrate; **c–f.** Conidiophores and conidiogenous cells; **g–j.** Conidia; **k, l.** Surface and reverse view of colonies on PDA after 36 days of incubation at 25 °C. Scale bars: 30 μm (**c–f**); 5 μm (**g–j**).

#### Culture characteristics.

Conidia produce germ tubes on PDA within 14 hours of incubation at 25 °C. Colonies on PDA are circular, with a flat surface and entire edge, reaching a diameter of 26 mm after 36 days of incubation at 25 °C. The colony appears dark brown to black at centre, brown at outer ring on the top view and brown on the reverse view.

#### Material examined.

China • Guizhou Province, Guiyang City, Qingzhen City, Maige Miao and Bouyei Ethnic Township, Xiaochong Village, 26°74′N, 106°37′E, on the rotting wood of *Phellodendron amurense*, 1 October 2024, Shi-Ping Zou, HB-Z17-1 (HKAS 145879, holotype), ex-type culture GZCC 25-0005 • *Ibid*., HB-Z17-2 (HKAS 145878), living culture GZCC 25-0006.

#### Notes.

Morphological examination indicated that *Helicosporium phellodendri* conforms to the generic concept of *Helicosporium*, characterised by pale yellow to yellow-green colonies on the natural woody substratum, erect setiferous cylindrical conidiophores with denticulate conidiogenous cells and hyaline to yellow-green helicoid conidia which are mainly becoming loosely coiled in water (Ma et al. 2024b). *Helicosporium phellodendri* is morphologically similar to *H. multidentatum*, but differs in having longer conidia (105–130 μm vs. 60–65 μm), with more coils (3¼–3¾ vs. 2½–3). Furthermore, the conidia of *H. multidentatum* become loosely coiled in water, while those of *H. phellodendri* do not loosen (Xiao et al. 2023). Phylogenetic analysis (Fig. 1) also supports that *H. phellodendri* and *H. multidentatum* are distinct species. Strains GZCC 25-0005 and GZCC 25-0006 formed a well-supported, independent lineage within *Helicosporium* (Fig. 1). *Helicosporium viridisporum and H. sexuale* are closely related to *H. phellodendri*. However, *H. viridisporum* can be distinguished from *H. phellodendri* by its longer conidiophores (69–189 × 3–5 μm vs. 89–97 × 4–5 μm), denticulate conidiogenous cells and acropleurogenous, aseptate conidia (Lu et al. 2022). *Helicosporium sexuale* displays sexual morph (Boonmee et al. 2021), whereas, *H. phellodendri* exhibits asexual morph. Therefore, *H. phellodendri* is introduced here as a novel species.

### ﻿ Neohelicomyces

Taxon classificationFungiTubeufialesTubeufiaceae

Z.L. Luo, Bhat & K.D. Hyde, Cryptog. Mycol. 38(1): 39 (2017)

98E12299-ADBF-5FF2-82E7-9876BBCF2FF2

#### Notes.

*Neohelicomyces* was proposed by Luo et al. (2017) with *N. aquaticus* designated as the type species. To date, 30 species have been recorded in Index Fungorum (2025). All known taxa represent asexual morphs (Ma et al. 2024a, b), except for *N. sexualis*, which was characterised and linked to its sexual morph, based on both morphological and molecular evidence (Sun et al. 2025). The asexual morphs of *Neohelicomyces* species are characterised by macronematous, mononematous, erect, septate conidiophores with holoblastic conidiogenous cells and loosely or tightly coiled helicoid conidia (Hsieh et al. 2021; Lu et al. 2022; Yang et al. 2023; Ma et al. 2024a, b). In contrast, the sexual morph produces scattered, superficial, solitary, subglobose, light brown ascomata on natural substrates, lacking setae. Ascospores are multiseriate, narrowly cylindrical, straight to slightly curved, hyaline to pale brown, septate and rough (Sun et al. 2025). Species of *Neohelicomyces* are saprobic, typically inhabiting freshwater or terrestrial environments on decaying wood in Asia, Europe and North America, including records from China, Germany and Thailand (Ma et al. 2024a; Sun et al. 2025). Only a few host plants have been documented for this genus (Ma et al. 2024a).

### ﻿ Neohelicomyces guizhouensis

Taxon classificationFungiTubeufialesTubeufiaceae

J. Ma, Y.Z. Lu & K.D. Hyde, MycoKeys 105: 323 (2024)

FAB80A6E-7F0B-5E3F-B38C-5FFF2F74DE23

901915

Fig. 4

#### Description.

*Saprobic* on dead wood of *Phellodendron amurense*. ***Sexual morph*** Undetermined. ***Asexual morph*** Hyphomycetous, helicosporous. ***Colonies*** on natural substrate superficial, effuse, gregarious, with mass of crowded conidia, white. ***Mycelium*** partly immersed, partly superficial, composed of hyaline to pale brown, branched, septate, guttulate, smooth hyphae. ***Conidiophores*** 154–175 × 4–5 μm (x̄ = 161 × 4.5 μm, n = 20), macronematous, mononematous, erect, cylindrical, tapering towards the apex, straight or slightly flexuous, unbranched, septate, subhyaline to pale brown, thick-walled. ***Conidiogenous cells*** 11–19 × 2.5–4 μm (x̄ = 14.5 × 3.5 μm, n = 20), holoblastic, monoblastic or polyblastic, integrated, terminal or intercalary, cylindrical, with denticles, subhyaline to pale brown, smooth-walled. ***Conidia*** solitary, acropleurogenous, helicoid, developing on tooth-like protrusion, 14–20 μm diam. and conidial filament 1.5–3 μm wide (x̄ = 16 × 2 μm, n = 20), 94–133 μm long (x̄ = 113 μm, n = 25), tightly coiled 3–3½ times, not becoming loose in water, guttulate, hyaline, smooth-walled.

**Figure 4.**
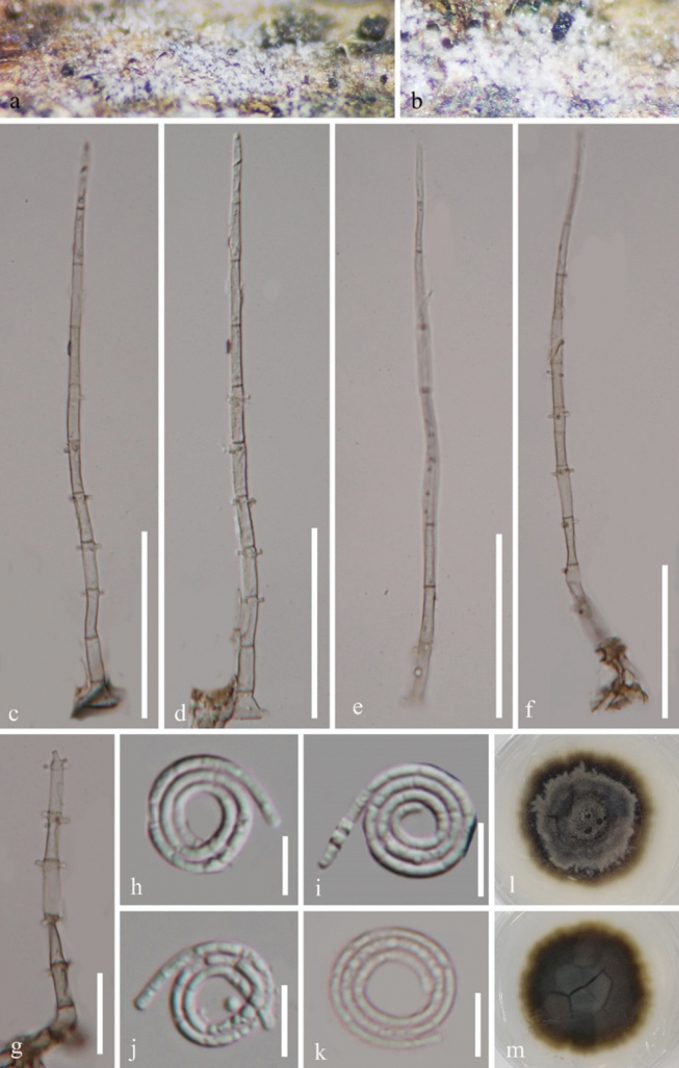
*Neohelicomyces guizhouensis* (HKAS 145881). **a, b.** Colonies on the natural substrate; **c–g.** Conidiophores; **h–k.** Conidia; **l, m.** Surface and reverse view of colonies on PDA after 45 days of incubation at 25 °C. Scale bars: 50 μm (**c–f**); 20 μm (**g**); 10 μm (**h–k**).

#### Culture characteristics.

Conidia produce germ tubes on PDA within 10 hours of incubation at 25 °C. Colonies on PDA are circular, with a raised centre and undulate edge, reaching a diameter of 30 mm after 45 days of incubation at 25 °C. The colony appears grey to pale brown on the top view, with the reverse side ranging from pale brown to brown.

#### Material examined.

China • Guizhou Province, Guiyang City, Qingzhen City, Maige Miao and Bouyei Ethnic Township, Xiaochong Village, 26°74′N, 106°37′E, on the rotting branch of *Phellodendron amurense*, 1 October 2024, Shi-Ping Zou, HB-Z15-1 (HKAS 145881), living culture GZCC 25-0007 • *Ibid*., HB-Z15-2 (HKAS 145880), living culture GZCC 25-0008.

#### Notes.

Phylogenetic analysis showed that our isolates (GZCC 25-0007 and GZCC 25-0008) grouped with *N. guizhouensis* (GZCC 23-0725) by strong support (ML/PP = 98%/0.97; Fig. 1). Pairwise comparison of the ITS, LSU, *tef*1-α and *rpb*2 sequences between GZCC 25-0007 and *N. guizhouensis* (GZCC 23-0725) revealed minimal nucleotide differences: 5 bp/424 bp (1.2%, including two gaps), 3 bp/859 bp (0.3%, including two gaps), 2 bp/892 bp (0.2%, no gaps) and 2 bp/903 bp (0.2%, no gaps), respectively. Morphologically, our new collections share identical characteristics of conidiogenous cells and conidia with the holotype of *N. guizhouensis* (Ma et al. 2024a). Therefore, GZCC 25-0007 and GZCC 25-0008 are identified as *N. guizhouensis*. *Neohelicomyces guizhouensis* was originally described by Ma et al. (2024a) from submerged wood in a stream in China and our study represents its first record associated with *Phellodendron amurense*.

### ﻿ Dictyosporium

Taxon classificationFungiPleosporalesDictyosporiaceae

Corda, Weitenweber’s Beitr. Nat. 1: 87 (1837)

27F8C3BB-10E6-5CCA-8810-F65D61BFCFFB

#### Notes.

*Dictyosporium* was proposed by Corda (1836) with *D. elegans* designated as the type species. Species of *Dictyosporium* are distributed across the globe in both freshwater and terrestrial habitats, functioning as saprobes and frequently found on decaying wood, plant litter,and in soil (Van Emden 1975; Boonmee et al. 2016; Yang et al. 2018; Zhang et al. 2023). To date, a total of 91 species has been recorded within this genus (Index Fungorum 2025).

### ﻿ Dictyosporium phellodendri

Taxon classificationFungiPleosporalesDictyosporiaceae

S.P. Zou, Y. P. Xiao & Y.Z. Lu sp. nov.

B02086B7-AC53-5678-B33B-6E7F31AC07BD

Index Fungorum: IF904506

Fig. 5

#### Etymology.

The specific epithet “*phellodendri*” refers to the host genus name “*Phellodendron*” from which the fungus was isolated.

#### Holotype.

HKAS 149888.

#### Description.

*Saprobic* on dead wood of *Phellodendron amurense*. ***Sexual morph*** Undetermined. ***Asexual morph*** Hyphomycetous. *Colonies* on natural substrate forming sporodochial conidiomata, superficial, punctiform, scattered, black, glistening. ***Mycelium*** immersed, pale to brown. ***Conidiophores*** 3–5 μm wide, micronematous, simple, aseptate, cylindrical, hyaline to pale brown, thin-walled. ***Conidiogenous cells*** 5–8 × 3–6 μm (x̄ = 6.3 × 4.7 μm, n = 10), holoblastic, hyaline or pale brown, cylindrical. ***Conidia*** 17–44 × 15–27 μm (x̄ = 30 × 21 μm, n = 30), acrogenous, cheiroid, complanate, smooth-walled, pale brown or pale yellow, mostly consisting of four closely adpressed rows of cells, rarely with three or five rows, 33–38 ×4–6 μm (x̄ = 35.5 × 5.5 μm, n = 30), 4–10-euseptate in each row, with hyaline, tubular, elongated apical appendages arising from two outer rows, 13–58 × 3–6 μm (x̄ = 32.9 × 4.2 μm, n = 15).

**Figure 5.**
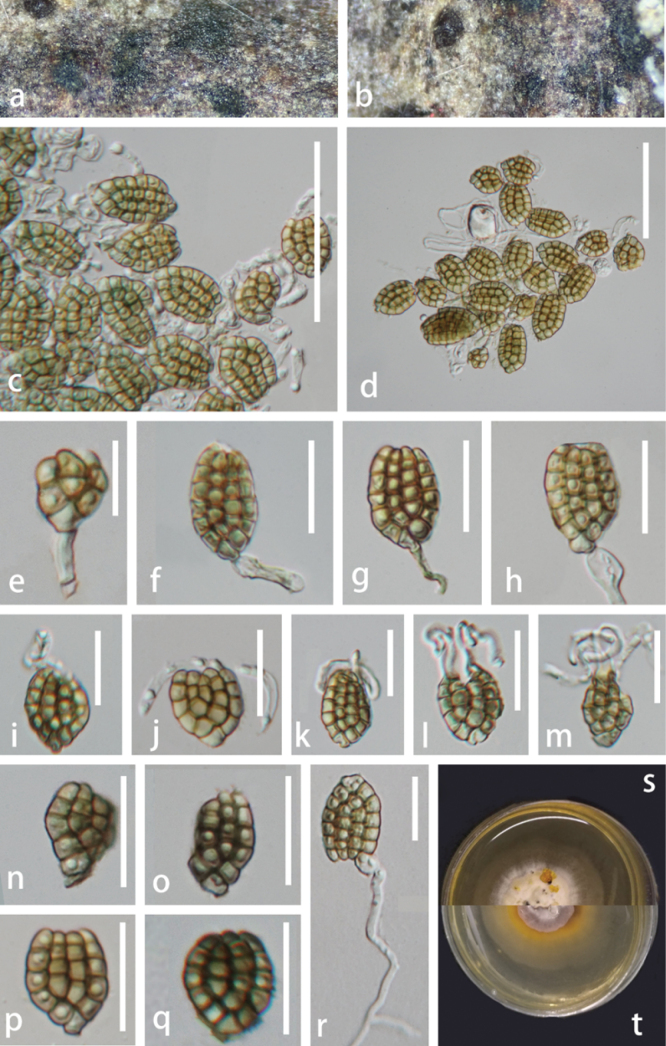
*Dictyosporium phellodendri* (HKAS 149888, holotype). **a, b.** Colonies on the natural substrate; **c, d.** Squash mount of a sporodochium; **e–h.** Conidia with conidiophores; **i–m.** Conidia with appendages; **n–q.** Conidia at different stages; **r.** Germinated conidium; **s, t.** Surface and reverse view of colonies on PDA after 39 days of incubation at 25 °C. Scale bars: 50 μm (**c, d**); 10 μm (**e**); 20 μm (**f–m**); 10 μm (**n**); 20 μm (**o–r**).

#### Culture characteristics.

Conidia produce germ tubes on PDA within 18 hours of incubation at 25 °C. Colonies on PDA are circular, with a flat surface and entire edge, reaching a diameter about 28 mm after 39 days of incubation at 25 °C. The colony appears white on the top view, while the reverse side with pale grey centre and yellow edge.

#### Material examined.

China • Guizhou Province, Guiyang City, Anshun City, Xixiu District, Yaowang Valley, 26°16'17"N, 106°7′0"E, on the rotting branch of *Phellodendron amurense*, 15 October 2024, Shi-Ping Zou, HBYWGC13 (HKAS 149888, holotype), ex-type culture GZCC 25-0522 • *Ibid*., HBYWGC25 (149889), living culture GZCC 25-0523.

#### Notes.

In the phylogenetic analysis, *Dictyosporium phellodendri* (GZCC 25-0522 and GZCC 25-0523) formed a sister clade to *D. muriformis* with 80% ML/0.98PP (Fig. 2), but *D. muriformis*, possesses smaller conidia (20–30 × 11–14.5 μm vs. 26–32 × 15–25 μm), darker pigmentation (median brown vs. pale brown or pale yellow) and the absence of appendages (Hyde et al. 2020). Morphologically, *D. phellodendri* is most similar to *D. alatum* (ATCC 34953) in conidial shape. However, it is distinguished by its larger conidia (17–44 × 15–27 μm vs. 26–32 × 15–25 μm), paler colouration and 4–10 septa per row (Van Emden 1975). Furthermore, comparisons of sequences between *D. phellodendri* and *D. alatum* (ATCC 34953) revealed differences in the ITS region of 13% (64/507 bp, including 11 gaps) and in the LSU region of 3% (18/573 bp, with 4 gaps). We, therefore, designated our collections as a new species, based on morphological and phylogenetic evidence.

## ﻿Discussion

In this study, three saprobic fungal taxa were collected from terrestrial habitats in Guizhou Province, China. Based on multigene phylogenetic analyses combined with morphological comparative evidence, *Helicosporium phellodendri* and *Dictyosporium phellodendri* are proposed as two novel species, while *Neohelicomyces guizhouensis* is identified as a new host record.

The family Tubeufiaceae (Tubeufiales, Dothideomycetes, Ascomycota) represents an important lineage of helicosporous fungi. Most genera and species in the family exhibit distinct asexual morphs, characterised by filamentous, helicoid (spirally coiled) conidia, which are key diagnostic features for species identification (Barr 1979). To date, 33 *Helicosporium* species, including the new species introduced herein, have been recognised (Bai et al. 2025), while 30 species of *Neohelicomyces* have been accepted (Ma et al. 2025). Members of both genera are commonly isolated from decaying wood or plant litter of unidentified hosts in freshwater and terrestrial habitats (Peng et al. 2025; Sun et al. 2025). Most known species are represented only by their asexual morphs; to date, four species of *Helicosporium* and one of *Neohelicomyces* have been reported in their sexual morphs (Brahmanage et al. 2017; Xiao et al. 2023; Sun et al. 2025).

*Dictyosporium* species play a crucial ecological role as decomposers of decaying wood and plant litter and are reported worldwide from both terrestrial and aquatic environments (Dubey 2022). Ho et al. (2002) investigated the seasonality and succession of fungi on submerged wood and found that *Dictyosporium* species occurred on diverse woody substrates in Tai Po Kau Forest Stream. Furthermore, *Dictyosporium* has been shown to enhance plant growth and resistance to pathogen infection (Peitl et al. 2020; Jiang et al. 2023). *Dictyosporium phellodendri*, isolated from decay branch of *Phellodendron amurense*, suggests that this fungal community plays a functional role in wood decomposition of *Phellodendron amurense* in the ecosystem. Future research may reveal that species of this genus participate in a broader range of ecological functions.

The members of Tubeufiaceae, especially helicosporous taxa, are known to produce diverse bioactive secondary metabolites (Lu et al. 2018b; Ma et al. 2024b). Likewise, *Dictyosporium* species has been recognised as sources of novel natural products (Tran et al. 2019, 2020). All strains isolated in this study originated from the dead branches of *P. amurense*, a medicinal plant rich in bioactive compounds, such as berberine, polyphenols, fibre, phytosterols and carotenoids. Extracts of this plant exhibit a wide range of pharmacological properties, including anti-inflammatory, antibacterial and anticancer activities and are used in the treatment of pneumonia, diarrhoea and circulatory disorders (Kim et al. 2011; Steinmann et al. 2012; Velmurugan et al. 2017; Wang et al. 2017; Sun et al. 2019; Balážová et al. 2022; Erhan et al. 2023; Feng et al. 2023). The isolation of these saprobic fungi from *P. amurense* not only expands our understanding of their biodiversity, but also provides valuable resources for exploring novel bioactive metabolites and elucidating the ecological interactions between fungi and medicinal host plants.

## Supplementary Material

XML Treatment for Helicosporium

XML Treatment for Helicosporium phellodendri

XML Treatment for Neohelicomyces

XML Treatment for Neohelicomyces guizhouensis

XML Treatment for Dictyosporium

XML Treatment for Dictyosporium phellodendri

## ﻿Additional information

### Use of AI

No use of AI was reported.

### Author ORCIDs

Shi-Ping Zou https://orcid.org/0009-0001-8515-6885

Yuan-Pin Xiao https://orcid.org/0000-0003-1730-3545

Qiu-Yuan Tang https://orcid.org/0009-0002-1823-0333

Yu-Bi Chen https://orcid.org/0009-0004-7584-4221

Yong-Zhong Lu https://orcid.org/0000-0002-1033-5782

Ning-Guo Liu https://orcid.org/0000-0002-9169-2350

Dan-Feng Bao https://orcid.org/0000-0002-5697-4280

Ratchadawan Cheewangkoon https://orcid.org/0000-0001-8576-3696

### Data availability

All of the data that support the findings of this study are available in the main text.
